# Overview of the CLEOPATRA Trial: Implications for Advanced Practitioners

**Published:** 2016-01-01

**Authors:** Karen Herold

**Affiliations:** Hoag Memorial Hospital Presbyterian–Women’s Health Institute, Newport Beach, California

Genomic biomarkers have long been known as predictors of pharmacokinetic differences among individuals that lead to differences in plasma drug exposure. Overexpression of the human epithelial growth factor receptor (HER2) proto-oncogene is seen in about 25% of human breast cancer and leads to a more aggressive tumor phenotype with a poor prognosis (Rexer & Arteaga, 2012). In recent years, this protein has become an important biomarker and a target for breast cancer treatment.

Trastuzumab (Herceptin), a monoclonal antibody against the HER2 receptor, was the first major breakthrough in the treatment of HER2-positive breast cancer (Oliveira, Braga, Passos-Coelho, Fonseca, & Oliveira, 2011). Monoclonal antibodies activate immune effectors to kill tumor cells through complement cascade or antibody-dependent cellular cytotoxicity (Shuptrine, Surana, & Weiner, 2012). Another effective monoclonal antibody is pertuzumab (Perjeta). Dual targeting with the monoclonal antibodies pertuzumab and trastuzumab plus chemotherapy was shown to increase median survival in the recently published Clinical Evaluation of Pertuzumab and Trastuzumab (CLEOPATRA) trial in the setting of HER2-positive metastatic breast cancer.

## Brief Overview of Breast Cancer

With a lifetime risk of approximately 12%, breast cancer is the most common cancer in women; nationwide, one in eight women will be diagnosed with breast cancer (National Cancer Institute, 2015; Howlader et al., 2012). Breast cancer remains the most frequent cause of cancer-related death in women, and although significant advances have been made in the treatment of breast cancer, many women still die of metastatic breast cancer (Ferlay, 2010). Each metastatic breast cancer presents a specific molecular profile and molecular mechanism of cancer progression, and although a large number of candidate targets for breast cancer treatment have been elucidated through genomic studies, only a few of them are validated as relevant and effective targets in clinical studies (Deluche, Onesti, & Andre, 2015).

Known risk factors for the development of breast cancer are varied and can include family history, genetic alterations, race, age, reproductive and menstrual histories, and breast density. Modifiable risk factors for breast cancer include hormone use, diethylstilbestrol (DES) use, alcohol consumption, physical activity, radiation therapy, and body weight (Steiner, Klubert, & Knutson, 2008). There are factors associated with breast cancer that are not clearly understood, and research continues to define more clearly the scientific basis and risk factors associated with breast cancer.

Breast cancers can be divided into two main types: carcinomas (cells that arise from the epithelial component of the breast) and sarcomas (cancers that arise from the stromal component of the breast; Johns Hopkins Medicine, 2015). Carcinomas are divided into invasive carcinoma and in situ carcinoma. Approximately 80% of breast carcinomas are invasive ductal carcinoma, and about 10% to 15% are invasive lobular carcinomas; it is important to distinguish between the various subtypes of cancer because they have different prognoses and treatment implications (Johns Hopkins Medicine, 2015).

## Human Epidermal Growth Factor Receptor

Estrogen receptor (ER), progesterone receptor (PR), and HER2/*neu* are the most important tissue markers in the management of metastatic breast cancer. Overexpression of HER2 is a negative prognostic factor in breast cancer and is found to be upregulated in 20% to 30% of all breast cancers; a patient diagnosed with HER2-positive breast cancer has more aggressive disease associated with an increased risk of metastases and shorter overall survival (Campiglio, Knyazev, & Ullrich, 1999; Gutierrez & Schiff, 2011).

Because HER2/*neu* proto-oncogene overexpression significantly contributes to the malignant development of breast cancer, molecular strategies that aim to downregulate this expression have become highly attractive approaches to treating breast cancer. Monoclonal antibodies directed toward the extracellular domain of HER2/*neu* have been developed and tested in clinical trials (Juntilla et al., 2009).

One successful example is trastuzumab, a humanized monoclonal antibody against the extracellular domain of the ~185 protein, which can cause downregulation of HER2/*neu*, and the advent of trastuzumab has become a milestone in the development of molecular targeted therapy (Dawood et al., 2008, Swain et al., 2014, Baselga et al., 2014). The HER2 gene expresses a cell surface receptor needed for cell growth, and trastuzumab is an antibody that blocks this cell surface receptor (Murck et al., 2015). Trastuzumab binds to the extracellular domain of HER2 and inhibits ligand-independent downstream oncogenic signaling (Scheuer et al., 2009).

Pertuzumab, another monoclonal antibody, is a HER2 dimerization inhibitor that binds to a different epitope on HER2 than trastuzumab and inhibits HER2 dimer formation with HER1, HER3, and HER4, which is required for HER2 to engender a signal (Scheuer et al., 2009). These complementary mechanisms of action led to the investigation of a comprehensive HER2 blockade, with a combination of anti-HER2 humanized monoclonal antibodies pertuzumab and trastuzumab. Although significant advances like these have been made with available HER2-targeted strategies, patients with HER2-positive breast cancer still faced the risk of progression in metastatic disease (Vu, Sliwkowski, & Claret, 2014).

Over the past 2 decades, the transmembrane tyrosine kinase (TKI) receptor HER2 (also known as ErbB2 or p185) has been shown to be an effective target for patients with breast cancer, but no treatment to effectively target the HER2 receptor has been particularly successful (Blok, Kuppen, van Leeuwen, & Sier, 2013). This changed recently when the CLEOPATRA trial met the a priori definition of success, with a promising 6-month progression-free survival (PFS) rate of at least 65% (Dang et al., 2015). The CLEOPATRA trial demonstrated superior PFS and overall survival when pertuzumab was added to trastuzumab and docetaxel in patients with HER2-positive metastatic breast cancer who had not received prior anti-HER2 therapy or chemotherapy for metastatic disease.

The addition of chemotherapy to trastuzumab and pertuzumab in the CLEOPATRA trial has resulted in a significant improvement in overall survival and PFS in patients with metastatic breast cancer. The CLEOPATRA trial had high-level outcomes, and now for the first time, we see an increased median survival that is unprecedented in first-line metastatic HER2-positive breast cancer. The CLEOPATRA trial is considered a landmark trial that has changed clinical practice. Dual targeting with pertuzumab and trastuzumab plus chemotherapy has now become the standard of treatment in metastatic breast cancer.

## Review of CLEOPATRA

The CLEOPATRA trial was presented at the 2011 San Antonio Breast Cancer Symposium and published that same day in *The New England Journal of Medicine*, with striking results of prolonged PFS in the metastatic setting of HER2-positive disease (National Institutes of Health [NIH], 2015). The CLEOPATRA trial showed that combining both HER2-targeting agents of trastuzumab and pertuzumab with docetaxel as initial treatment led to a 6-month improvement in PFS compared with trastuzumab, docetaxel, and placebo (NIH, 2015).

Baselga and colleagues (2012) implemented the CLEOPATRA trial to examine the effect of adding a second HER2 monoclonal antibody or placebo to docetaxel plus trastuzumab as initial treatment for HER2-positive metastatic breast cancer. CLEOPATRA was a large, randomized, double-blind, placebo-controlled, phase III study that recruited 808 patients from 204 centers across 25 countries. Adults who were at least 18 years old with locally recurrent, unresectable, or metastatic HER2-positive adenocarcinoma of the breast (without brain metastases), a normal left ventricular ejection fraction (≥ 50%), an Eastern Cooperative Oncology Group (ECOG) performance status (PS) of 0 or 1, and who had not received previous chemotherapy or anti-HER2 therapy for their metastatic disease were randomized to receive docetaxel, trastuzumab and pertuzumab or docetaxel, or trastuzumab and placebo.

For the final analysis of overall survival, it was determined that the study would have a power of 80% to detect a 33% improvement in the pertuzumab group (hazard ratio for death from any cause, 0.75) after the occurrence of 385 deaths, and the primary endpoint of the study was PFS based on tumor assessment (Swain et al., 2015). Review of PFS and objective response rates was stopped after this analysis. Secondary endpoints included overall survival, investigator-assessed PFS, and independent assessment of duration of response and safety.

**Results and Toxicity**

Results in the group receiving the pertuzumab combination were a median survival of 56.5 months (95% confidence interval [CI], 49.3 to not reached), compared with 40.8 months (95% CI = 35.8–48.3) in the group receiving the placebo combination—a difference of 15.7 months (hazard ratio favoring the pertuzumab group, 0.68: 95% CI = 0.56–0.84; *p* < .001) (Swain et al., 2015). Safety profiles including cardiac side effects were similar and consistent across both groups and time points (Swain et al., 2015).

Patients in the study could have received adjuvant/neoadjuvant chemotherapy with or without trastuzumab if ≥ 12 months had elapsed before diagnosis of metastatic disease; they were treated every 3 weeks until disease progression or dose-limiting toxicities (Baselga et al., 2012). Patients were accrued to the study from February 2008 to May 2010; 406 were randomly assigned to the control group (placebo plus trastuzumab plus docetaxel) and 402 were assigned to the treatment group (pertuzumab plus trastuzumab plus docetaxel). Baseline characteristics were similar in both groups. The rates of PFS, overall survival, and objective response were analyzed in all randomized patients. The log-rank test was used to compare PFS between the control and the treatment groups, and Kaplan-Meier analysis was used to estimate median PFS.

In the initial interim analysis report the median PFS was 12.4 months in the control group and 18.5 months in the pertuzumab group (hazard ratio for progression or death, 0.62; 95% CI = 0.51–0.75; p < .001; Baselga et al., 2012). The investigators reported similar adverse effects in both groups, with no increase in left ventricular systolic dysfunction; febrile neutropenia and grade ≥ 3 diarrhea were more common in the pertuzumab group (Baselga et al., 2012).

The second interim CLEOPATRA analysis was reported after the data cutoﬀ point (May 14, 2012), at which time 267 patients had died: 154 (38%) of the patients were from the placebo group and 113 (28%) were from the pertuzumab group (Swain et al., 2013). Median follow-up was 30 months in both groups, and overall survival was reported as 37.6 months (95% CI = 34.3–not estimable) in the placebo group and had not been reached (95% CI = 42.4–not estimable) in the pertuzumab group (hazard ratio 0.66, 95% CI = 0.52−0.84; *p* = .0008), which represented a signiﬁcant survival beneﬁt for patients who received pertuzumab along with trastuzumab plus docetaxel. Similar to the primary analysis, serious adverse events of febrile neutropenia, neutropenia, diarrhea, pneumonia, and cellulitis were experienced by 115 (29%) of the patients in the control group (placebo, trastuzumab, and docetaxel) and 148 (36%) in the group who received pertuzumab, trastuzumab, and docetaxel (Swain et al., 2013).

**Critical Questions and Study Limitations**

The most recent analysis of the CLEOPATRA data was reported by Swain and colleagues (2015) to answer two critical questions: (1) Does adding a second, different monoclonal antibody for HER2 to trastuzumab plus docetaxel chemotherapy increase overall survival in patients who have metastatic, HER2-positive breast cancer without increasing the burden of treatment (adverse effects)? (2) In first-line treatment of patients with metastatic, HER2-positive breast cancer, does adding pertuzumab to trastuzumab and docetaxel as compared to the addition of placebo significantly improve median survival?

Investigators answered the first question by showing that pertuzumab was safe, even when administered for long periods; while on study, the patients in the pertuzumab group received a median of 24 cycles (range, 1–96), and control group patients received 15 cycles (range, 1–67). Most adverse events occurred during docetaxel treatment, but an important finding was that pertuzumab did not increase long-term cardiac toxicity compared with placebo (Table 1).

**Table 1:**
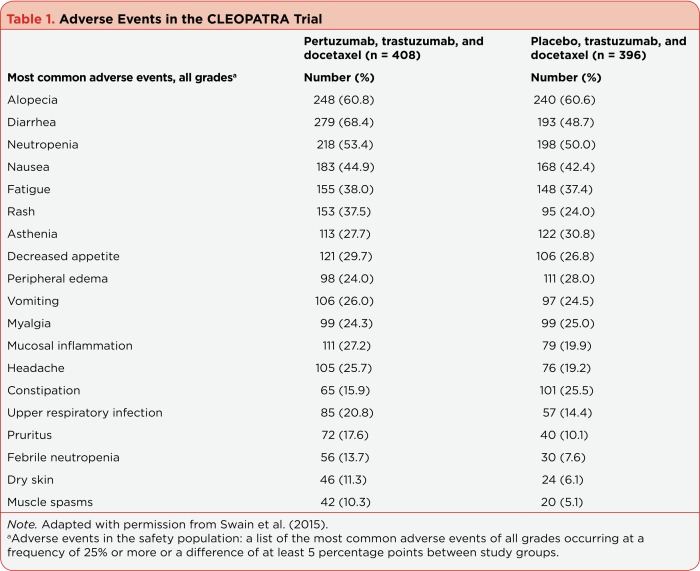
Adverse Events in the CLEOPATRA Trial

The second question was answered based on the last interim analysis, when investigators found that the addition of pertuzumab did increase survival: Median overall survival in the pertuzumab/trastuzumab/docetaxel group was 56.5 months (95% CI = 49.3–not estimable), as compared with 40.8 months (95% CI = 35.8–48.3) in the trastuzumab, docetaxel group (hazard ratio favoring the pertuzumab-containing regimen, 0.68; 95% CI = 0.56–0.84; p < .001) or 15.7 months longer (Table 2); see the related article by Dudley et al. starting on page 91 for the survival curves.

**Table 2:**
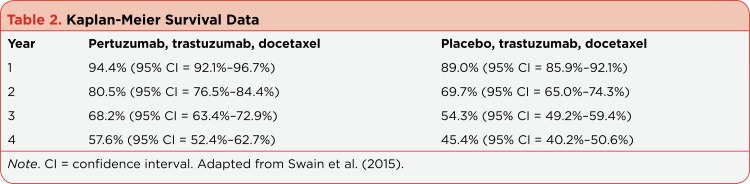
Kaplan-Meier Survival Data

To increase the survival benefit in the control group, between July and November 2012, 48 (11.8%) patients in the control group crossed over to receive pertuzumab (Swain et al., 2015). In these patients, the median PFS improved by 6.3 months, and the median duration of response improved by 7.7 months.

Of the original 808 patients in this study, 389 (48.1%) had died by this analysis: 168 of 402 (41.8%) pertuzumab-treated patients and 221 of 406 (54.4%) control group patients (hazard ratio favoring the pertuzumab group, 0.68; 95% CI = 0.56–0.84; p < .001). Median follow-up was 49.5 months (range, 0–70) in the pertuzumab group and 50.6 months (range, 0–69) in the control group. Overall survival rates were calculated by Kaplan-Meier estimates at the end of years 1 to 4 (Table 1). When crossover patients were excluded from analysis, median overall survival estimates were more conservative: 56.5 months (95% CI = 49.3 to not estimable) in the pertuzumab group and 34.7 months (95% CI = 31.2–39.4) in the control group.

Swain and colleagues (2015) addressed limitations and remaining important questions about the treatment of HER2-positive breast cancer. For instance, subgroup analysis of patients with nonvisceral disease was limited by small numbers, and thus the study had insufficient power for meaningful statistical interpretation. The authors were clear that most deaths during the CLEOPATRA trial were related to breast cancer, and they mentioned that better treatments are still needed.

The investigators agreed that more work needs to be done to answer important questions related to breast cancer treatment, including the best, most effective drug combinations and the length of drug treatment, especially in metastatic disease. They mentioned that this is especially important in early breast cancer treatment; the ongoing Adjuvant Pertuzumab and Herceptin in Initial Therapy of Breast Cancer (APHINITY) trial will assess the safety and efficacy of pertuzumab in addition to chemotherapy plus trastuzumab as adjuvant therapy in patients with operable HER2-positive primary breast cancer.

The authors were not clear whether hormonal treatment plus trastuzumab and pertuzumab is more effective in hormone receptor–positive disease than hormonal treatment plus trastuzumab alone and noted that there is no biomarker to predict which patients with HER2-positive disease would benefit most from pertuzumab and trastuzumab. In addition the authors agreed that it is not known whether chemotherapeutic agents other than docetaxel would improve the safety profile of the CLEOPATRA trial, because a phase II study assessing PFS at 6 months with pertuzumab, trastuzumab, and paclitaxel showed few grade 3 and 4 events and no cardiac toxicity. And, finally, the question of when, if ever, therapy with pertuzumab plus trastuzumab for metastatic breast cancer should be discontinued remains unanswered.

## Implications for Advanced Practice Providers 

Personalized medicine aims to increase the efficacy of therapeutics in disease and disease prevention by utilizing targeted therapy for breast cancer. The many types of breast cancer lead to the complexity of breast cancer treatment; as high-level evidence from studies emerge, more accurate biomarker treatments will become available.

Among newly diagnosed patients with breast cancer, it is important to know whether a breast tumor overexpresses HER2, so appropriate targeted therapy can be administered. Immunohistochemistry (IHC) and fluorescence in situ hybridization (FISH) assays test breast tumor tissue for overexpression or amplification of HER2 (Carlson, 2008). Many laboratories screen cases with IHC and triage select cases for FISH testing or use FISH as the only method for HER-2/*neu* testing, but the superiority of one method over another remains controversial (Ferretti, Felici, Papaldo, Fabi, & Cognetti, 2007).

This controversy has continued even after the 2007 publication by the American College of Clinical Oncology (ASCO) and the College of American Pathologists (CAP) entitled "Guideline Recommendations for Human Epidermal Growth Factor Receptor 2 Testing in Breast Cancer." Although the authors of the guideline concurred that about 20% of current HER2 testing may be inaccurate, they pointed out that HER2 status should be determined for all invasive breast cancers (Wolff et al., 2007). Cases with a diffuse intense circumferential membrane staining pattern in > 30% of the tumor (scored as 3+) should be considered positive by IHC, and when using FISH, HER2 positivity is defined as a HER2 gene copy > 6 or a HER2/CEP17 ratio > 2.2 (Hicks & Schiffhauer, 2011; Vranic et al., 2011). ASCO and CAP both recommend a testing algorithm that relies on accurate, reproducible assay performance and defines positive, equivocal, and negative values for both HER2 protein expression and gene amplification (Wolff et al., 2007). Furthermore, the panel strongly recommends the validation of laboratory assay or modifications, use of standardized operating procedures, compliance with new testing criteria to be monitored with the use of stringent laboratory accreditation standards, proficiency testing, and competency assessment. The panel recommends that HER2 testing be done in a CAP-accredited laboratory (Wolff et al., 2007).

##  Conclusion

Personalized treatment of breast cancer will continue to evolve in the 21st century as biomarkers continue to become elucidated and validated through clinical trials like the CLEOPATRA study. The development of technologies and methodologies in personalized medicine focusing on improving patient selection and detecting target engagement will significantly improve the morbidity and mortality that are associated with breast cancer in the 21st century. In clinical practice, we will increasingly see that it is the biology of the tumor that drives the choice of therapy.
